# Nanoparticle-Based Rice Husk Liquid Smoke as Periodontitis Therapy through OPG, RANK, and RANKL Expression

**DOI:** 10.1155/2024/5015893

**Published:** 2024-06-14

**Authors:** Ira Arundina, Theresia Indah Budhy, Aqsa Sjuhada Oki, Meircurius Dwi Condro Surboyo, Arvind Babu Rajendra Santosh, Sidarningsih, Indeswati Diyatri, Tytania Rahmaputry, Arya Pradana, Mohammad Iqbal, Azzahra Salsabila Adira Moelyanto

**Affiliations:** ^1^Department of Oral Biology, Faculty of Dental Medicine, Universitas Airlangga, Surabaya 60132, Indonesia; ^2^Department of Oral Pathology and Maxillofacial, Faculty of Dental Medicine, Universitas Airlangga, Surabaya 60132, Indonesia; ^3^Department of Oral Medicine, Faculty of Dental Medicine, Universitas Airlangga, Surabaya 60132, Indonesia; ^4^School of Dentistry, Faculty of Medical Sciences, University of the West Indies, Kingston, Jamaica; ^5^Faculty of Dental Medicine, Universitas Airlangga, Surabaya 60132, Indonesia; ^6^Dental Health Science, Faculty of Dental Medicine, Universitas Airlangga, Surabaya 60132, Indonesia

## Abstract

**Introduction:**

Periodontitis therapy employing nanomaterials with submicron sizes holds promise for enhancing osteogenesis and facilitating periodontal cell proliferation. This study aims to assess the potential of nanoparticle-based rice husk liquid smoke (*n*-RHLS) in an animal model of periodontitis by evaluating the expression of osteoprotegerin (OPG), receptor activator of nuclear factor-k*β* (RANK), and receptor activator of nuclear factor-k*β* ligand (RANKL).

**Methods:**

Twenty-eight male Wistar rats were inoculated with 10^9^ CFU/ml of *Porphyromonas gingivalis* in the sulcus mandibular incisor region to create periodontitis and subsequently treated with n-RHLS while the control with saline. Immunohistochemical staining was performed on the mandibular incisor to assess OPG, RANK, and RANKL expression 2 and 7 days after treatment.

**Results:**

OPG expression exhibited a significant increase at both 2 and 7 days, while RANKL expression decreased notably after 7 days of treatment using n-RHLS (*p* < 0.05). In contrast, RANK expression did not show significant differences compared to the control groups (*p* > 0.05).

**Conclusion:**

Nanostructured liquid smoke derived from rice husk nanoparticles (*n*-RHLS) demonstrates potential as a therapeutic agent for periodontitis, especially on OPG/RANK/RANKL expression, by modulating OPG and RANKL expression to support periodontal tissue health.

## 1. Introduction

Periodontitis is an inflammatory disease of the periodontal tissue primarily caused by specific bacteria found in subgingival plaque, which triggers an inflammatory response in the gingiva [1]. One of the key culprits of infection, *Porphyromonas gingivalis*, produces various products, including extracellular vesicles, enzymes (collagenases, proteases, and hyaluronidases), toxins (e.g., leukotoxin), and metabolites (such as hydrogen sulfide), all of which can disrupt periodontal tissue [2]. The primary approach to treating periodontitis involves eliminating the causal bacterial infection and preventing further destruction [3]. The mainstay of periodontal disease therapy involves scaling and root planning procedures, often with or without surgical interventions [4]. However, as the pathogenic microbiota in periodontal tissue becomes more complex over time, additional therapy with local and systemic antibiotics and anti-inflammatories becomes necessary to control bacterial infections and tissue damage [5]. Comprehensive periodontitis therapy aims to achieve bone and connective tissue reconstruction, including regeneration, repair, or the formation of new attachments [6].

Traditional medicine therapies utilizing natural ingredients are commonly practiced in Indonesia, with rice being one such plant with medicinal potential. One of the medicinal plants that were explored for its medicinal potential was rice husk. Rice husks can be processed into liquid smoke suitable for healthcare applications. Liquid smoke is produced through pyrolysis and contains organic components such as phenol and acetic acid [7]. Liquid smoke derived from rice husk offers numerous health benefits, including blood glucose reduction in diabetics [8], antioxidant properties [9], antibacterial effects [10], and anti-inflammatory properties [9].

To optimize the therapeutic potential of active compounds in liquid smoke derived from rice husk, efficient delivery to therapeutic targets is essential. Nanoparticle technology, involving materials with dimensions typically ranging from 1 to 100 nm, can facilitate this goal [11]. Nanoparticle-based preparations offer several advantages, including enhanced absorption, increased bioavailability, a high surface area, and the ability to influence microenvironments in targeted tissues. These properties can promote osteogenesis and facilitate the absorption of proteins essential for neomatrix formation, thereby supporting cell attachment and proliferation [12]. Liquid smoke derived from rice husk shows promise as a candidate for drug development due to its anti-inflammatory properties, which can modulate osteogenesis [13] by inhibiting proinflammatory cytokines [14, 15]. When formulated as nanoparticles, substances with smaller sizes allow for efficient cellular uptake [16]. The preceding investigation revealed that the n-RHLS possesses an average particle size of approximately 33 nm. These nanoparticles demonstrated a notable stimulatory effect on osteoblast proliferation [13] and concurrently exhibited inhibitory properties against *Porphyromonas gingivalis*, a bacterium implicated in the pathogenesis of periodontitis [17]. Given these promising outcomes, there is a compelling rationale to advance the investigation to an in vivo model. This step aims to substantiate the potential efficacy of the n-RHLS in mitigating periodontitis.

The primary target in the treatment of periodontitis is the prevention of further alveolar bone destruction by addressing the balance of OPG, RANK, and RANKL [18]. These markers are crucial for understanding the mechanisms underlying bone loss and tissue destruction, evaluating disease severity, assessing treatment outcomes, and exploring potential therapeutic interventions to restore periodontal tissue health [19]. Previous studies have shown that liquid smoke from rice husk has the potential to inhibit *Porphyromonas gingivalis* [14, 20], maintain the viability of osteoblasts [13], and suppress proinflammatory cytokines in periodontitis [15]. In nanoparticle form, it will be more effective for *Porphyromonas gingivalis* and decrease the inflammatory response and the RANKL expression. OPG acts as a regulatory factor, modulating the RANKL/RANK interaction and influencing bone regeneration. In light of this fact, the transformation of liquid smoke into n-RHLS has the potential to affect periodontitis by enhancing the expression of OPG, RANK, and RANKL, making it a promising candidate for periodontitis therapy.

## 2. Materials and Methods

### 2.1. Animal Model

In this experimental study, male Wistar rats aged five months were utilized as the animal model. A total of twenty-eight rats were employed and categorized into four groups based on differing treatment regimens and observation durations. The research protocol received ethical approval from the Institutional Ethics Committee under registration number 580/HRECC.FODM/XI/2021.

The sample size for each treatment group was determined based on the Lameshow sample size formula, with an *α* value of 1.92 and an *β* value of 0.84. The minimum required sample size for this study was established as three.

### 2.2. Periodontitis Model and Treatment with *n*-RHLS

Each mouse received an injection of *Porphyromonas gingivalis* bacteria (Pg ATCC 33277) at a concentration of 1 × 10^9^ colony-forming units (CFU) in a 20 *μ*l phosphate-buffered saline (PBS) solution, administered using a 0.5 CC syringe into the gingival sulcus on the mesial side of the lower mandibular central incisor [14, 15]. Bacterial injections were administered once every three days over a two-week period. Clinical signs of periodontitis were assessed on the 14th day postbacterial induction, and these signs included reddening of the gingiva, interdental swelling in the mandibular incisor region, and evidence of attachment loss upon probing, indicative of the presence of a periodontal pocket [21] (Supplementary Materials 1). For the control group, sterile water was applied, while the experimental group received *n*-RHLS, both applied to the gingival sulcus on the mesial side of the lower mandibular central incisor for 2 and 7 days [15].

### 2.3. OPG, RANK, and RANKL Expression

Mandibular tissue samples were fixed in 10% neutral buffered formalin for 24 hours and subsequently decalcified using EDTA over a 30-day period. Following decalcification, the tissues were embedded in paraffin blocks and sectioned into 4-*μ*m-thick slices using a microtome. Antigen retrieval was achieved by incubating the tissue sections in a citrate buffer (pH 6.0) at 37°C for 24 hours. Blocking was performed with 5% bovine serum for 30 minutes at room temperature. Primary antibodies used included OPG (rabbit polyclonal, 1 : 50, antibody online, antibodies-online Inc., Germany), RANK (rabbit polyclonal, 1 : 50, antibody online, antibodies-online Inc., Germany), and RANKL (rabbit polyclonal, 1 : 200, antibody online, antibodies-online Inc., Germany), with an overnight incubation at 4°C. Visualization of protein expression was achieved using diaminobenzidine for peroxidase-conjugated secondary antibodies. Expression levels were quantified by counting positive staining in the alveolar bone area in the central incisive mandibular (as a region of interest). Five different regions of interest were selected and analysed in 1000x magnification using a light microscope.

### 2.4. Statistical Analysis

The expression data for each parameter were presented as the mean ± standard deviation. Data normality was assessed using the Shapiro–Wilk test, and homogeneity was checked using the Levene test. To evaluate the differences in the expression of OPG, RANK, and RANKL between the 2-day and 7-day groups, two-way ANOVA was conducted with Tukey's HSD post hoc. Statistical significance was defined as *p* < 0.05 and analysed using PRISM software (PRISM 9 for macOS, GraphPad, Boston, USA).

## 3. Results

### 3.1. *n*-RHLS Elevated OPG Expression

**Figure 1:**
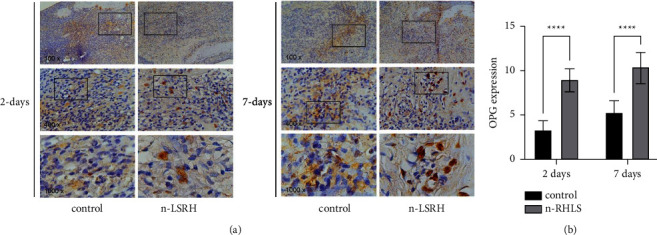
Treatment with n-RHLS elevated the OPG expression in the alveolar bone area in the central incisive mandibular. (a) Immunohistochemistry staining for OPG expression. (b) Quantification of OPG expression. Data are means ± S.D. (error bars) (*n* = 3 areas per group) (one-way ANOVA and Tukey's posttest) ^*∗∗∗∗*^*p* < 0.0001, with the indicated groups.

Figure 1(a) shows the OPG expression in the alveolar bone area in the central incisive mandibular. OPG expression was significantly higher in both time observations compared to the control groups. Treatment with *n*-RHLS resulted in significantly elevated OPG expression compared to the control group (*p* < 0.001) (Figure 1(b)).

### 3.2. *n*-RHLS Not Affected RANK Expression

**Figure 2:**
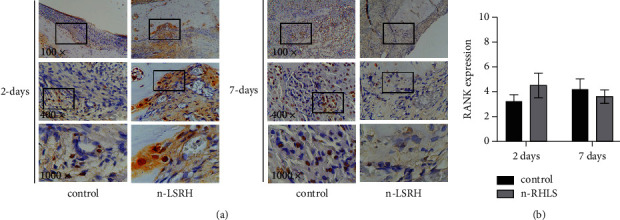
Treatment with n-RHLS elevated the RANK expression in the alveolar bone area in the central incisive mandibular. (a) Immunohistochemistry staining for RANK expression. (b) Quantification of RANK expression. Data are means ± S.D. (error bars) (*n* = 3 areas per group) (one-way ANOVA and Tukey's posttest).

Figure 2(a) shows the RANK expression in the alveolar bone area in the central incisive mandibular. Treatment with n-RHLS showed comparable RANK expression to the control (Figure 2(b)).

### 3.3. *n*-RHLS Decreased RANKL Expression

**Figure 3:**
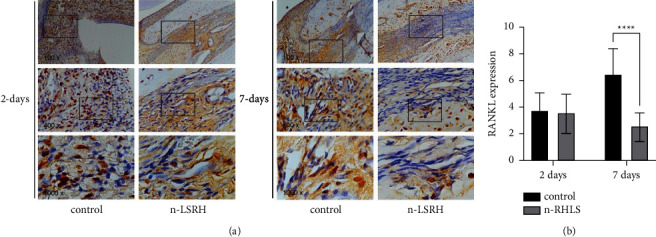
Treatment with *n*-RHLS elevated the RANKL expression in the alveolar bone area in the central incisive mandibular. (a) Immunohistochemistry staining for RANKL expression. (b) Quantification of RANKL expression. Data are means ± S.D. (error bars) (*n* = 3 areas per group) (one-way ANOVA and Tukey's posttest) ^*∗∗∗∗*^*p* < 0.0001, with the indicated groups.

Figure 3(a) shows the RANKL expression in the alveolar bone area in the central incisive mandibular. Treatment with n-RHLS led to lower RANKL expression compared to the control group, but this difference was significant only at 7 days, with no significant difference observed at 2 days (Figure 3(b)).

## 4. Discussion

The transformation of liquid smoke into nanoparticles or nanoencapsulation represents a novel advancement in harnessing liquid smoke [22, 23]. Nanoparticle or nanoencapsulation technology for liquid smoke has been employed for its original purpose, which is as a food preservative [24, 25], as well as for its antimicrobial properties in food [26], and antioxidant potential [27]. In terms of therapeutic effects, rice husk liquid smoke has been successfully transformed into *n*-RHLS, demonstrating antibacterial activity against bacteria implicated in periodontitis [17] and the ability to stimulate osteoblast formation [13].

In our study, we harnessed the potential of rice husk liquid smoke converted into nanoparticles, leveraging its rich compound composition. This liquid smoke predominantly contains compounds such as guaiacol and phenols [28, 29], which are known for their anti-inflammatory and antioxidant properties. Specifically, guaiacol and phenols can inhibit COX-2, contributing to the overall mitigation of inflammation [30, 31]. Furthermore, guaiacol, present in n-RHLS, possesses the ability to reduce oxidative stress, regulate inflammatory mediators, and stimulate osteoclast differentiation and activation by modulating the production of key signalling proteins, such as OPG, RANK, and RANKL [32, 33]. Our findings, supported by the data, underscore the significant impact of these polyphenols on OPG, RANK, and RANKL expression, exemplifying the remodelling process within inflammatory tissues. Polyphenols from n-RHLS promote osteoblast proliferation, leading to an increased OPG ratio and the regulation of osteoblast differentiation markers [34].

The observed upregulation of OPG expression following treatment with n-RHLS is of particular interest, as OPG serves as a decoy receptor for RANKL, thereby inhibiting RANKL-induced osteoclast activation and bone resorption [35]. The significant increase in OPG expression suggests that n-RHLS may enhance the protective mechanisms against excessive bone resorption in the alveolar bone area in the central incisive mandibular, potentially contributing to improved periodontal health. The lack of a significant impact on RANK expression suggests that treatment with n-RHLS may not directly influence the formation of osteoclasts in this context. Instead, the potential modulation of the OPG/RANKL ratio by *n*-RHLS may play a more critical role in regulating bone remodelling in the alveolar bone area in the central incisive mandibular.

The observed decrease in RANKL expression at 7 days following treatment with n-RHLS may be indicative of a delayed response or a time-dependent effect. Further investigations are warranted to elucidate the underlying mechanisms governing this temporal variation in RANKL expression. In conclusion, our study provides valuable insights into the impact of treatment with n-RHLS on the OPG/RANK/RANKL axis in the alveolar bone area in the central incisive mandibular. The significant upregulation of OPG expression suggests a potential protective role of n-RHLS in preventing excessive bone resorption, while the nuanced effects on RANK and RANKL expression warrant further investigation. These findings contribute to our understanding of alveolar bone remodelling and may have implications for oral health interventions. Understanding the impact of *n*-RHLS on the OPG/RANK/RANKL axis enhances our comprehension of alveolar bone remodelling dynamics. This knowledge can guide clinicians in developing targeted approaches to maintain or restore alveolar bone health. The treatment with *n*-RHLS may potentially contribute to strategies for preventing or minimizing bone loss in periodontitis. Further research is needed to explore the precise mechanisms through which *n*-RHLS modulates these molecular players and its clinical relevance.

One limitation of this study lies in its exclusive reliance on tissue analysis of OPG, RANK, and RANKL expression in the periodontitis following treatment with *n*-RHLS. Future research should consider analysing the markers related to bone regeneration like osteoclast activity using TRAP staining, and alkaline phosphatase expression as a marker of osteoblast. The purpose of analysing osteoblast and osteoclast activity is to complement the assessment of OPG, RANK, and RANKL expression in tissue, providing a comprehensive understanding of the dynamic interplay within the bone microenvironment. Examining osteoblast and osteoclast function offers insights into the downstream effects of the OPG/RANK/RANKL axis, contributing to a more holistic characterization of bone remodelling processes and potential implications for therapeutic interventions.

## 5. Conclusion

Nanostructured liquid smoke derived from rice husk nanoparticles (*n*-RHLS) demonstrates potential as a therapeutic agent for periodontitis, especially on OPG/RANK/RANKL expression, by modulating OPG and RANKL expression to support periodontal tissue health.

## Data Availability

All the data are available upon personal request to the corresponding author (ira-a@fkg.unair.ac.id).

## Supplementary Materials

Supplementary Materials Clinical signs (redness and swelling) of periodontitis postbacterial induction in the central incisive mandibular.
