# Supplementary material for: Nanoparticle-Based Rice Husk Liquid Smoke as Periodontitis Therapy through OPG, RANK, and RANKL Expression
Source: Int J Biomater. 2024 Jun 14;2024:5015893. doi: 10.1155/2024/5015893 (PMC11192596; doi:10.1155/2024/5015893)
Supplement: Supplementary Materials — Clinical signs (redness and swelling) of periodontitis postbacterial induction in the central incisive mandibular. [file 5015893.f1.docx]

**Supplementary file**

**Nanoparticle-based rice husk liquid smoke as periodontitis therapy through OPG, RANK and RANKL expression**

**Supp Table 1.** Clinical signs of periodontitis post-bacterial induction in the central incisive mandibular

| Group assignment | Sample number | Redness | Swelling |
| --- | --- | --- | --- |
| control | 1 | + | + |
|  | 2 | + | + |
|  | 3 | + | + |
|  | 4 | + | + |
|  | 5 | + | + |
|  | 6 | + | + |
|  | 7 | + | + |
|  | 8 | + | + |
|  | 9 | + | + |
|  | 10 | + | + |
|  | 11 | + | + |
|  | 12 | + | + |
|  | 13 | + | + |
|  | 14 | + | + |
| nLSRH | 15 | + | + |
|  | 16 | + | + |
|  | 17 | + | + |
|  | 18 | + | + |
|  | 19 | + | + |
|  | 20 | + | + |
|  | 21 | + | + |
|  | 22 | + | + |
|  | 23 | + | + |
|  | 24 | + | + |
|  | 25 | + | + |
|  | 26 | + | + |
|  | 27 | + | + |
|  | 28 | + | + |

*+ = present the sign*
